# A study of the relationship between characteristic traits and Employee Engagement (A case study of nurses across Kermanshah, Iran in 2015)

**Published:** 2015

**Authors:** A Ziapour, N Kianipour

**Affiliations:** * Social Development & Health Promotion Research Center, Kermanshah University of Medical Sciences, Kermanshah, Iran,; ** Research Committee, Kermanshah University of Medical Sciences, Kermanshah, Iran

**Keywords:** Staff Engagement, personality traits, nurses, Kermanshah

## Abstract

Staff Engagement is an individual’s interest and enthusiasm to accomplish the specified duties, all together with his sustained profession with organizations. Accordingly, the current research aimed to delve into the relationship between the characteristical traits and Staff Engag ement among nurses employed in Kermanshah-based hospitals in 2015. In this descriptive-correlational study, 322 nurses of public hospitals in Kermanshah were picked in 2015. For information gathering, Schaufeli & Bakker’s Utrecht Staff Engagement scale and NEO Five-Factor Inventory (NEO-FFI) were used. Information was examined through descriptive analytics (Frequency, Rate, Average, and Standard Deviation) and inferential analytics (Pearson Correlation Test and Multiple Regression Analysis). Also, the 21st version of SPSS software was applied for information investigation. The results demonstrated that the greatest and smallest means of characteristical traits among nurses related to acceptance to experience (3.75 ± 0.63) and neuroticism (2.82 ± 0.55). Also, the highest and lowest means of Staff Engagement related to absorption (5.41 ± 0.76) and vigor (5.04 ± 0.86). Moreover, the outcomes of the Pearson correlation examination showed that there were important connections between the two dimensions of personality traits, i.e. neuroticism (P<0.001, r=0.172) and extraversion (P<0.001, r=0.038), and job engagement. Moreover, neuroticism had the most meaningful relationship with Staff Engagement (P<0.001, r=0.172). On the other hand, the outcomes of multiple regression analysis revealed that dutifulness and agreeableness were good predictors for job engagement. Given that the two scopes of personality traits, i.e. dutifulness and agreeableness, were closely related to work engagement, it was suggested that these dimensions were given a careful consideration in the event of employing workforce, especially nurses, with the aim of boosting the organizational productivity.

## Introduction

Personality is a complicated psychological construct used for the discovery of the way individuals behave and as a general rule, shows different kinds of human behaviors in diverse situations [**44**]. The individuals’ personality dimensions fall into different categories. The five factors of neuroticism (including traits such as nervousness, moodiness, and tempera mentality), extraversion (implying an energetic approach and includes traits such as sociability, activity, assertiveness, and positive emotionality), acceptance to experience (including traits such as imagination, curiosity, and creativity), agreeableness (including traits such as altruism, tender-mindedness, trust, and modesty), and dutifulness (including traits such as organization, thoroughness, and reliability) were introduced by Costa & McCrae (1992) as basic biological inclinations [**10**]. These basic inclinations are readiness to act and feel in certain manners and are not directly affected by the environment [**48**]. According to this model, individuals can adopt certain inclinations and attitudes towards tasks and goals in organizations based on their personality traits [**46**]. Thus, the distinctions in the individuals’ personality traits can be a source of creativity or the root cause of hassles in organizations, and they can influence actions, conducts, and decisions across enterprises [**47**]. Since personality traits act as factors that determine the individuals’ behaviors, identifying such traits can result in increasing the Staff Engagement in organizations through predicting behaviors [**35**,**44**]. Given that the variable of Staff Engagement is a new, positive idea, it has proven to be capable of suitably predicting occupational and personal outcomes [**7**,**17**]. In recent years, Staff Engagement has interested so many scholars and has been given a great deal of attention. Staff Engagement is broadly accepted to predict staffs' outcomes, departmental progress, and commercial achievement [**8**,**17**,**30**,**36**]. In fact, Worker Engagement is an effective construct with high potential in identifying helpful departmental outcomes. It is the individuals' attention and willingness to accomplish the specified functions, together with their extended profession with departments, and the flow of such energy across departments, working as an extra-energy inside individuals, which is useful to work crowds and the entire society [**9**]. William Kahn (1990), as the first scholar of this field, defined “work engagement” as “using the whole of one’s being to carry out the assigned job-related roles”. To perform the assigned roles in work engagement, individuals use or express their whole physical, cognitive, and emotional traits. In the absence of work engagement, individuals are physically, cognitively, and emotionally detached from their job-related roles [**22**,**30**]. 

The surveys conducted by consulting companies revealed that the level of the employees’ Staff Engagement is taking a downward trend [**12**,**29**]. Moreover, most of the institutions are suffering from a lack of work engagement, which has appeared in the imposition of sky-high costs on organizations [**29**]. Some positive attachments are created between staffs and organizations through work engagement, having positive consequences for both sides, including positive, strong occupational attitudes towards jobs, mental and psychological health (e.g., positive emotions and reductions in burnout), better job performance, enhancing intrinsic motivation, adopting personal initiatives and proactive behaviors, and acquisition of occupational and personal interests [**23**,**41**]. In the presence of Staff Engagement and loyalty in organizations, an intimate and familiar atmosphere is created, as a result of which work processes get facilitated, and the pace of the work groups’ actions get adjusted and advance with a full throttle towards the set aims [**41**]. In studies conducted by Cheung (2008), the results demonstrated that such factors as communication and conducts mixed with trust, accountability and clear duties, sufficient number of qualified nurses, conversant, proficient, reliable leadership, collaborative decision-making, understanding the value of nurses’ jobs, and the chance of professional growth, play vital roles in the staffs’ continuation of employment with organizations [**49**]. Haslam et al. (2003) have the idea that no emotional connections in institutions, important contacts, meaningful planning, and administration exist without Staff Engagement [**18**]. The outcomes of investigations completed by González et al. (2005) showed that personality traits had significant relationships with age and gender, and examinations conducted by Halbesleben (2010) revealed that there was a positive connection between work capacity and Staff Engagement (vigor and dedication) [**16**]. The outcomes of the examinations managed on nurses by Jahanbakhsh Ganjeh et al. (2009) demonstrated that they had the highest scores in titles of dutifulness and flexibility [**21**]. "Work engagement" is marked as "positive, satisfying, job-related mental states that are differentiated by three indexes of vigor, dedication, and absorption" [**38**].

Since nurses are seen as the most prominent resources in hospitals, they make quite an impact on the levels of health and hygiene in societies through the agency of delivering diverse health care services [**7**,**11**,**32**]. The differences in personality traits among nurse communities influence their manner of Staff Engagement and interactions with patients and are of vital significance [**5**,**34**]. Staff Engagement is necessary for all occupations, including nursing jobs and helps managers assess the degree of the employees’ personality traits and loyalty to organizations.

Therefore, given that no earlier researches have dealt with the connection between the nurses' personality traits and job engagement, the current research directed to investigate the foregoing. Moreover, regarding the meaning and different reality of the scopes of the work engagement, the connection between personality traits and work engagement, and their effects are ambiguous, so explaining such a connection is considered a scientific need. 

## Methodology

The current research is a descriptive-correlational study. According to the statistics collected from the HRM Department of the Medical School of Kermanshah in 2014, the statistical community consisted of all nurses employed in public hospitals all over Kermanshah (n=1987, including 1542 males and 445 females). Also, the number of the sample community was determined through Cochran’s sample size formula (n=322, including 249 females and 73 males), chosen by the agency of stratified-random sampling. For data gathering, three applications were applied (1) a demographic application including the staffs' individual data (gender, lifetime, training, work experience, and career positions), (2) NEO Five-Factor Inventory (NEO-FFI) [**10**], and (3) Schaufeli & Bakker’s Utrecht Staff Engagement scale [**38**]. 

The NEO Five-Factor Inventory (NEO-FFI) was based on the factor investigation and was constructed by Costa & McCrae (1992) in Baltimore, Maryland in 1985. This questionnaire consisted of 60 questions with five-point Likert scaling (1 = strongly disagree, 2 = disagree, 3 = neutral, 4 = agree, 5 = strongly agree) and examined the five scopes of personality traits: acceptance to participation, responsibility, extraversion, agreeability, and neuroticism [**37**,**15**]. The content validity of NEO Five-Factor Inventory (NEO-FFI) was confirmed by Costa & McCrae (1992), and the reliabilities of neuroticism, extraversion, acceptance to experience, agreeability, and dutifulness were 0.90, 0.78, 0.76, 0.86 and 0.90, respectively [**10**]. In Iran, the five-factor structure of this questionnaire was generally confirmed by Garousi Farshi et al. (2001), and their internal consistency reliability coefficients were reported by the measure of Cronbach’s alpha as 0.86, 0.73, 0.56, 0.68 and 0.87, respectively [**13**]. In the American sample, the reliabilities of neuroticism, extraversion, acceptance to experience, agreeability, and dutifulness were 0.93, 0.87, 0.76, 0.89, and 0.86, respectively [**1**]. In a study conducted by Kiamehr (2002), the internal coherence reliability coefficient of this application was reported by the measure of Cronbach's alpha (ranging from 0.54 to 0.79) [**24**]. Furthermore, in studies performed by Hejazi & Iravani (2002), the Cronbach’s alpha for this questionnaire was 0.74 [**19**]. In the present study, Cronbach’s alphas of neuroticism, extraversion, agreeability, acceptance to experience, and dutifulness were 0.91, 0.78, 0.76, 0.73, and 0.86, respectively.

As for the Staff Engagement application, it was created by Schaufeli et al. (2002) and involved in 17 inquiries with seven-point Likert Scaling (0=strongly disagree,1=quite disagree, 2=slightly disagree, 3=neither, 4=slightly agree, 5=quite agree, 6=strongly agree) and analyzed 3 scopes of energy, devotion, and intake [**38**]. This application has been used in China, Finland, Greece, Japan, South Africa, and Spain and has a high trustworthiness and soundness [**39**]. The content soundness of Staff Engagement inquiry has been verified in examinations conducted by Abaszadeh et al. (2013) and Isakhani et al. (2013) [**2**,**3**]. In the current research, the Cronbach's alphas of vigor, dedication, and absorption were 0.92, 0.89, and 0.86, sequentially. As for data examination, the definitive statistics (Frequency, Rate, Average, and Standard Deviation) and inferential statistics (Pearson Correlation Examination and Multiple Regression Study) were employed. Also, the 21st version of SPSS software was applied for data examination. 

## Results

Out of the 322 members in the existing investigation, 73 people (22.7%) were males and 249 individuals (77.3%) were females. The Average and Standard Deviation of the ages of the sample population were 31.54 ± 6.03. The 31-40-year-old age group held the biggest number (125 individuals, 38.8%). In titles of training, 260 individuals (80.7%) held a B.A. and 62 individuals (19.3%) held an M.A. and higher degrees. Most samples (115 individuals, 35.7%) had 21-26 years of job background. The Mean and Standard Deviation of the work background of the sample community were 18.48 ± 6.5. In titles of work posts, most samples (235 individuals, 73%) were head nurses (see **Table 1**).

As for the scopes of personality traits in the sample community, the outcomes showed that the acceptance to experience sustained the greatest level (Mean=3.75, SD=0.63) and neuroticism held the lowest level (Mean=2.82, SD=0.55) (see **Table 2**). 

As for the scopes of Staff Engagement in the sample community, the outcomes showed that absorption held the greatest degree (Mean=5.41, SD=0.76) and vigor took the smallest degree (Mean=5.04, SD=0.86) (see **Table 2**).

The outcomes of the Pearson correlation coefficient examination revealed that there were certain, notable connections between the two scopes of personality traits, i.e. neuroticism (p<0.001, r=0.172) and extraversion (p<0.001, r=0.038) and work engagement. Furthermore, neuroticism and extraversion had the biggest and smallest connections with work engagement, sequentially (**Table 3**).

Concerning the connections between the five scopes of personality traits (acceptance to experience, dutifulness, extraversion, agreeability, and neuroticism) and work engagement, the outcomes of stepwise linear regression tests indicated that only two dimensions of Dutifulness and agreeability remained in the final model, and the other scopes were eliminated. Given the β coefficients, dutifulness and agreeableness significantly specified work engagement. Also, examining the standard coefficients showed that dutifulness (β =0.148) and agreeableness (β =0.140) had the biggest and the smallest impacts on the depending variable of nurses' work engagement, respectively (**Table 4**).

**Table 1:** The Demographic Characteristics of participants

Variables	Groups	No (%)
Gender	male	73 (22.7%)
	female	249 (77.3%)
Age (years)	30≥	171 (53.1%)
	31-40	125 (38.8%)
	41-50	26 (8.1%)
Education	Bachelor’s degree	260 (80.7%)
	Master’s Degree	62 (19.3%)
Work Experience (years)	5≥	29 (9%)
	6-10	24 (7.5%)
	11-15	16 (5%)
	16-20	110 (34.2%)
	26-21	115 (35.7%)
	26≤	28 (8.7%)
Positions	nurse managers	22 (6.8%)
	Supervisor	65 (20.2%)
	Head nurse	235 (73%)

**Table 2:** The Average, Standard Deviation, Lowest Score, Highest Score and participants' Rankings

Statistical indexes	Variables	Average	SD	Rank
Personality traits	Acceptance to experience	3.75	0.63	First
	Extroversion	3.48	0.52	Second
	Dutifulness	3.46	0.39	Third
	Agreeableness	3.18	0.32	Fourth
	Neuroticism	2.82	0.55	Fifth
	Personality traits	3.34	0.29	-
Employee Engagement	(Absorption)	5.41	0.76	First
	(Dedication)	5.24	0.82	Second
	(Vigor)	5.04	0.86	Third
	Staff Engagement	5.23	0.48	-

As for the independent variable of personality traits, the results showed that acceptance to experience held the highest mean score (Mean=3.75, SD=0.63) and neuroticism held the lowest mean score (Mean=2.82, SD=0.55) (see **Table 2**). In total, the Average and Standard Deviation of nurses' personality traits were 3.34 and 0.29, respectively, which indicated that all indexes of nurses' personality traits were at average levels. As for the independent variable of work engagement, the results showed that absorption held the highest mean score (Mean=5.41, SD=0.76) and vigor held the smallest average amount (Mean=5.04, SD=0.86) (see **Table 2**). In total, the Average and Standard Deviation of nurses' Staff Engagement were 5.23 and 0.48, respectively.

**Table 3:** Pearson correlation coefficients between nurses’ personality traits and work Engagement

Hypothesis	Independent variable	Dependent variable	Connection coefficient	Sig (2-tailed)
1	Neuroticism	work Engagement	0.172**	0.002
2	Agreeableness	work Engagement	0.165**	0.003
3	Dutifulness	work Engagement	0.072	0.199
4	Acceptance to experience	work Engagement	0.061	0.276
5	Extroversion	work Engagement	0.038	0.496
6	(Total) Personality traits	(Total) work Engagement	0.162**	0.004
**Connection is significant at 0.01 (2-tailed)				

As it was shown in **Table 3**, there was a positive, direct, significant connection between the nurses’ personality traits and Staff Engagement (p<0.001, r=0.162). Except for the dutifulness that had a negative, insignificant relationship with work engagement, the other ones (openness to experience, extraversion, agreeableness, and neuroticism) had positive, direct, significant relationships with Staff Engagement (p<0.001). In addition, neuroticism had the most important relationship with Staff Engagement (p<0.001, r=0.172), while extraversion had the least important relationship with Staff Engagement (p<0.001, r=0.038).

To predict the level of Staff Engagement based on different dimensions of personality traits, the stepwise multiple regression examination was used. The results of this test indicated that the two dimensions of Neuroticism and Agreeableness predicted 4.9% of the variance of nurses’ work engagement. Therefore, after eliminating the insignificant variables (openness to experience, extraversion, and dutifulness), the outcomes of multiple regression examination for predicting the nurses’ Staff Engagement happened as it follows (see **Table 4**):

**Table 4:** The outcomes of multiple regression test for predicting the nurses’ work Engagement

The scopes of personality traits	Unstandardized Coefficients		R	R Square	Standardized Coefficients	t	Sig.
	B	Std. Error			Beta		
(Constant)	4.197	0.279	0.221	0.049		15.067	0.000
neuroticism	0.128	0.048			0.148	2.681	0.008
Agreeableness	0.211	0.083			0.140	2.533	0.012

## Discussion 

The current research aimed to delve into the connection between personality traits and Staff Engagement among the nurses of hospitals placed in Kermanshah in 2015. The outcomes of the existing research revealed that the Average and Standard Deviation of nurses' personality traits were 3.34 and 0.29, respectively. This outcome was coherent with the outcomes of examinations carried out by Zaidi et al. (2013), Ziapour et al. (2015), and Inceoglu & Warr (2012) [**20**,**43**,**45**]. The maximum and minimum levels of personality traits related to the acceptance to experience and neuroticism, respectively. To put it bluntly, the less the neuroticism developed in nurses, the more the Staff Engagement increased, while the more the acceptance to experience developed in nurses, the more their Staff Engagement was. One explanation for the foregoing might be that the ones with high levels of neuroticism are incapable of coping with conflicts and anxieties, resulting in a lack of Staff Engagement in the workplace. To some extent, this outcome was coherent with the outcomes of examinations carried out by Komarraju et al. (2009), Zhang & Bruning (2011), and Naseh et al. (2012) [**4**,**26**,**32**,**44**]. In examinations completed by Langelaan et al. (2006), the outcomes showed that the Staff Engagement was differentiated by low levels of neuroticism and high levels of extraversion [**27**]. In studies carried out by Kim et al. (2009) and Neetu (2013), the results showed that the Staff Engagement could be predicted by neuroticism and dutifulness [**25**,**33**]. 

The outcomes of the existing research revealed that the Average and Standard Deviation of the nurses’ Staff Engagement were 5.23 and 0.48, respectively. Also, the maximum and minimum levels of Staff Engagement related to absorption and vigor. The results of the studies conducted by Abaszadeh et al. (2013) revealed that the Average of nurses' Staff Engagement in Sirjan-located hospices was 3.50 out of 6. In investigations conducted by Mauno et al. (2007) [**31**], the Average of nurses' Staff Engagement in hospices was 4.45 out of 6. Furthermore, in American samples, the results of the studies carried out by Lawarence (2009) [**28**] revealed that the Average of nurses' Staff Engagement in American hospices was 4 out of 6, which was lower than the mean of the Staff Engagement in the existing research. 

There was a meaningful correlation between neuroticism and work engagement in the existing research, which was coherent with the outcomes of investigations performed by Swider & Zimmerman (2010), Shimizutani et al. (2008), Azeem (2010), and Ghorpade et al. (2007) [**6**,**14**,**40**,**52**]. Due to such symptoms such as anxiety, insecurity, and anger in nurses with high degrees of neuroticism, it was expected that these staffs had high degrees of job engagement, too. Therefore, given the tendency of these staffs towards negative feelings, it was anticipated that neurotic individuals had higher degrees of job engagement. 

In addition, there was a notable correlation between agreeableness and work engagement, which was to some extent coherency with the outcomes of investigations performed by Swider & Zimmerman (2010), Shimizutani et al. (2008), Azeem (2010), and Ghorpade et al. (2007) [**6**,**14**,**40**,**42**]. Those with great degrees of agreeability, even when working in horrible conditions, do their utmost to adapt to their working conditions and their agreeableness (which involves traits such as altruism, tender-minded, trust, and modesty) gives them enough incentive to achieve personal success. Agreeable individuals are cooperative, reliable people. Therefore, their perceptions of work performance in the future should result in positive psychological conditions. In the existing research, no relationships were found between the three scopes of personality traits, i.e. dutifulness, acceptance to experience and extraversion, and the dependent variable of work engagement. 

The current research was faced with several limitations: (1) information were gathered throughout self-reporting method, which may affect the correctness of outcomes, (2) as a result of the fact that the information was gathered from subjects in Kermanshah-located hospitals, the outcomes cannot be established to other nurses employed in other hospitals across Iran. Given the prominent roles that nurses' personality traits play in hospitals, it is recommended that further studies are conducted in other governmental and private hospices throughout Iran and the results are compared with one another.

## Conclusion 

The maximum and minimum levels of personality traits related to the acceptance to experience and neuroticism, respectively. Furthermore, two dimensions of work engagement, i.e. absorption and vigor, held the highest and lowest means among staffs. Out of five dimensions of personality traits, the two variables of neuroticism and agreeableness had significant relationships with Staff Engagement and could specify it. The individuals' personality traits, as inherent components of human beings' personalities, influence organizational environments. The organizational commitment, one result of the outcomes of work engagement, is directly influenced by the individuals' personality traits, deemed the biggest human resources in hospitals influencing the level of services outstandingly. Therefore, to increase the productivity of human resources in organizations, especially for major managerial positions, it is recommended that the individuals’ personality traits are given acute consideration in the event of recruitment and appointment of the workforce. 

**Acknowledgements**

This article was according to the findings of the study plan No. 93375, Aided by the Vice Chancellery of Research & Technology of the Medical School of Kermanshah. In the end, the kindly supports and assistance of the rector of the Medical School of Kermanshah and our colleagues are sincerely appreciated.
